# Biochar Implications Under Limited Irrigation for Sweet Corn Production in a Semi-Arid Environment

**DOI:** 10.3389/fpls.2022.853746

**Published:** 2022-04-22

**Authors:** Manpreet Singh, Sukhbir Singh, Ved Parkash, Glen Ritchie, Russell W. Wallace, Sanjit K. Deb

**Affiliations:** ^1^Department of Plant and Soil Science, Texas Tech University, Lubbock, TX, United States; ^2^Department of Crop and Soil Sciences, University of Georgia, Tifton, GA, United States; ^3^Texas A&M AgriLife Research and Extension, Lubbock, TX, United States

**Keywords:** water productivity, water holding capacity, drought stress, semi-arid, physiology, photosynthesis, plant available water

## Abstract

The integration of biochar and deficit irrigation is increasingly being evaluated as a water-saving strategy to minimize crop yield losses under reduced irrigation in arid and semi-arid regions such as West Texas. A 2-year (2019 and 2020) open-field study evaluated the effect of two types of biochar amendments (hardwood and softwood) and three irrigation rates [100, 70, and 40% crop evapotranspiration (ET_*c*_) replacement] on the physiology, plant growth, and yield of sweet corn in semi-arid West Texas. All experimental units were replicated four times in a split-plot design. The chlorophyll content (Chl_*SPAD*_) in 40% ET_*c*_ dropped significantly compared to 100% ET_*c*_ and 70% ET_*c*_ during the reproductive phase. Although water stress under 40% ET_*c*_ decreased photosynthesis (*P*_*n*_) to limit transpiration (E) by stomatal closure, it improved intrinsic water use efficiency (iWUE). The above-mentioned gas exchange parameters were comparable between 100% ET_*c*_ and 70% ET_*c*_. Both biochar treatments increased Chl_*SPAD*_ content over non-amended plots, however, their effect on gas exchange parameters was non-significant. All growth and yield-related parameters were comparable between 100% ET_*c*_ and 70% ET_*c*_, but significantly greater than 40% ET_*c*_, except water productivity (WP). Both deficit irrigation treatments improved WP over full irrigation in 2019, but in 2020, the WP gains were observed only under 70% ET_*c*_. Hardwood biochar decreased soil bulk density and increased soil porosity, but it had a marginal effect on the water retention characteristics. These results suggest that 70% ET_*c*_ can be used as an alternative to full irrigation to save water with a minimal yield penalty for sweet corn production in the West Texas region. The hardwood biochar application improved the vegetative biomass significantly but yield marginally during the first 2 years of application. A long-term study is required to test the effect of biochar under deficit irrigation beyond 2 years.

## Introduction

The High Plains region of Texas in the United States is water-stressed like many arid and semi-arid regions of the world (Nielsen-Gammon et al., 2020). Global climate change, increasing population, and water use have exacerbated the uncertainty of water availability in the future and increased the vulnerability to drought events. Drought is the most critical abiotic stress, which impairs the plant’s physiological processes, growth, and causes heavy yield losses (Seleiman et al., 2021). Therefore, the availability of an irrigation source is necessary to ensure future food security. The average yearly reference evapotranspiration (ET_*o*_) of 1,500 mm in the Lubbock County of West Texas is higher than the 469 mm of average rainfall (TAMU, 2021). This necessitates the need for supplemental irrigation to meet crop water requirements and maintain high-crop yields. The Ogallala aquifer is the primary source of water on the Texas High Plains and more than 90% of the water derived from this aquifer is being used for irrigation purposes (McGuire, 2017). The high water extraction rate has caused a serious decline in the water levels, especially in West Texas (Daher et al., 2019). Hence, strategies promoting the efficient use of irrigating water are required to sustain the water resources and ensure food security in the future.

Crop yields are generally the highest under optimal water application, but crops can adapt and produce reasonably under limited water (FAO, 2002). Deficit irrigation (DI), a strategy of applying less water than the evapotranspiration demands, is generally employed to increase water productivity (WP). However, water stress can induce several physiological and biochemical changes in the plant, ultimately affecting its morphology. Water stress reduces stomatal conductance (*g*_*s*_), which in turn moderates the transpiration (E) and leaf gas exchange (Seleiman et al., 2021). Transpiration plays an important role in regulating metabolic activities by moderating the leaf temperature (Sterling, 2005). The reduction in gas exchange decreases CO_2_ assimilation. The loss of turgor due to moisture stress retards cell elongation and division causing reduced leaf expansion. Thus, water stress can adversely affect net photosynthesis by reducing the leaf-level photosynthesis (*P*_*n*_) and decreasing the leaf area. The reduction in net photosynthesis ultimately reduces biomass production and yield. However, plants can adjust to a maintain high *P*_*n*_ with a moderate reduction in the *g*_*s*_, and *P*_*n*_ is generally less sensitive to water stress than *g*_*s*_ (Liu et al., 2005; Pazzagli et al., 2016; Parkash, 2020). Thus, the leaf-level intrinsic water use efficiency (iWUE), defined as the ratio of *P*_*n*_ and *g*_*s*_, is expected to increase under moderate water stress. It is important to quantify iWUE gains at different DI levels and evaluate if these are translated into WP at the crop level. A few researchers have investigated the feasibility of DI in sweet corn (*Zea mays* L. var. *rugosa*) in terms of yield and biomass production (Stone et al., 2001; Farsiani et al., 2011; Saberi et al., 2012; Motazedian et al., 2019) but studies assessing the DI effects on sweet corn physiology are lacking.

Sweet corn (*Zea mays* L. var. *rugosa*) is a warm-season crop relished for its tastefulness, high-sugar concentration, soft kernels, and thin shell (Oktem et al., 2003). Sweet corn is considered sensitive to water stress due to its shallow root system (Laboski et al., 1998). Oktem (2008) observed a 6, 22, and 37% decline in the sweet corn yield with 10, 20, and 30% reduction in the irrigation compared to full irrigation, respectively. Ertek and Kara (2013) suggested that 85% of full irrigation could serve as an alternative without a significant decrease in the sweet corn yield. These conflicting results suggest that water stress can lead to dramatic fluctuations in the sweet corn yield, especially in drought-prone semi-arid regions like the Texas High Plains.

The plant responses to water stress vary with climate and soil (Singh et al., 2021). Soil organic amendments have been suggested to improve the soil’s physiochemical and microbial properties (Ding et al., 2016; Blanco-Canqui, 2017; Atkinson and Aitkenhead, 2018). Biochar, a carbon-rich product of pyrolysis of organic matter is increasingly being studied as a soil amendment to mitigate drought stress. Previous literature suggests that biochar application generally decreases the bulk density, and increases soil porosity and water retention (Blanco-Canqui, 2017), although responses vary with biochar feedstock, pyrolysis conditions, and soil type (Spokas et al., 2012).

Reviewing the available reports, Blanco-Canqui (2017) noted an increase in plant available water with biochar application in 72% of the cases. Several recent studies revealed the improvement in yield and WP of various crops with integrated use of biochar application and DI (Ali et al., 2018; Faloye et al., 2019; Singh et al., 2019; Alfadil et al., 2021). In a greenhouse tomato experiment, using 25 t ha^–1^ wheat straw biochar, Agbna et al. (2017) observed significant improvement in transpiration and photosynthesis rate and obtained comparable yield between water-stressed and full irrigation treatments. Gavili et al. (2019) observed an increase in stomatal conductance, water use efficiency (WUE), growth, and yield of greenhouse-grown drought-stressed soybean plants treated with 1.25% (w/w) cattle manure biochar compared to untreated plants. Similarly, an open-field study conducted by Langeroodi et al. (2019) suggests that soil amendment using 10 t ha^–1^ maize straw biochar improved chlorophyll content, seed yield, and WUE of pumpkin subjected to DI on a silt loam soil in a semi-arid climate. Contrarily, Ramlow et al. (2019) observed no significant effect of broadcasting 25 t ha^–1^ woody biochar on biomass and yield of maize under full or limited irrigation. Liu et al. (2017) observed a negative effect of 0.74% (w/w) birch wood biochar on biomass and WUE of potato plants raised in pots filled with sandy loam soil. The results obtained in the above-mentioned studies with different biochars in various crops subjected to DI suggest that biochar may influence crop physiology and growth depending on factors like biochar feedstock and preparation procedure, crop type, soil characteristics, and climatic conditions. Therefore, investigation of such effects and the mechanisms involved is necessary before implementing the use of any biochar material in crop production.

Currently, no report investigating the use of biochar for mitigating water stress in sweet corn is available in the literature. Field studies investigating the interactive effects of DI and biochar application on crop plants are lacking in semi-arid regions like the Texas High Plains. We hypothesized that using DI in sweet corn can save water, and biochar can alleviate the negative effect of water stress and help maintain crop productivity. The combined use of DI and biochar can be a part of water and food sustainability approaches needed in the semi-arid Texas High Plains. The objective of this study is to evaluate the effect of two biochar types and DI levels on physiology, growth, yield, and WP of sweet corn.

## Materials and Methods

### Site Description

The field experiments were conducted in 2019 and 2020 at the Quaker Research Farm, Texas Tech University, Lubbock, TX (33° 36′ 18″ N, -101° 54′ 26″W, and 992 m above sea level). The trials were carried out on the same experimental plots during both years. The climate of the experimental site is semi-arid with an average annual rainfall of 469 mm, mostly concentrated from May to October. The average annual high and low temperatures are 23.3 and 7.8 °C, respectively. The average annual evapotranspiration is 1,501 mm, far exceeding the average annual rainfall (TAMU, 2021). The soil of the experimental site is described as Amarillo sandy clay loam (fine-loamy, mixed, super active, thermic Aridic Paleustoll).

### Land Preparation and Planting

The seedbeds were prepared with a tractor-mounted disk plow. The seeds of a sweet corn hybrid, Remedy, were planted using a four-row planter at the rate of 7.4 kg/ha maintaining a 100 cm spacing between the rows. The planting was done on 5 May 2019 and 2020. However, in 2020, replanting was done on 15 May due to poor germination. The plant density obtained after crop establishment was 4.1 and 3.3 plants m^–2^ in 2019 and 2020, respectively. Mechanical weeding was performed once when the crop was in the knee-high stage in both years. Thereafter, manual weeding was done as needed. The field was irrigated using a subsurface drip irrigation system laid at the depth of 30 cm under each bed and 100 cm apart. All experimental plots received equal amounts of fertilizer based on the soil test recommendations. The fertilizers were applied through a drip irrigation system with 112 kg N/ha at 4 weeks after sowing and 56 kg N/ha at 8 weeks after planting in 2019, and 6 weeks after planting with 90 kg N/ha in 2020 using URAN 32 (32-0-0, Nitrogen Fertilizer Solution, Nutrien Ag Solution, Loveland, Colorado).

### Biochar Properties and Application

Two types of biochar prepared from different feedstock, hardwood-oak, and softwood-pine used in this experiment were acquired from Wakefield™ BioChar. The hardwood biochar was prepared through slow pyrolysis at 350°C for 24 h whereas the softwood biochar was prepared at 500°C for 15 min. The physical and chemical characteristics of the two biochars are described in Table 1. Both biochars were spread in the respective field plots at 13 Mg ha^–1^ and incorporated into the soil using a tractor-mounted rotary tiller once on 9 April 2019, approximately 1 month before sowing.

**TABLE 1:** Characteristics of hardwood and softwood biochars.

Characteristics	Hardwood-oak	Softwood-pine
Total organic matter	82.07 % wt.	95.12 % wt.
Total carbon	62.96 % wt.	88.01 % wt.
Total ash	17.93 % wt.	4.88 % wt.
pH	8.6	7.4
Nitrogen	0.64 % wt.	0.59 % wt.
Total phosphate	3.52 mg/kg	4.53 mg/kg
Potassium	2,960 mg/kg	614 mg/kg
Sulfur	0.011 % wt.	0.031 % wt.
Hydrogen	2.09 % wt.	0.40 % wt.
Oxygen	16.37 % wt.	6.09 % wt.
Calcium	64,900 mg/kg	4,128 mg/kg
Copper	1.72 mg/kg	3.57 mg/kg
Iron	1,770 mg/kg	595 mg/kg
Magnesium	4,540 mg/kg	1,225 mg/kg
Manganese	1,040 mg/kg	234 mg/kg
Zinc	23.2 mg/kg	4.59 mg/kg
Surface area correlation	17.74 m^2^/g	375.76 m^2^/g

### Experimental Design and Treatments

A split-plot design was used for randomizing the irrigation and biochar treatment combinations. Three irrigation treatments, 100, 70, and 40% of crop evapotranspiration (ET_*c*_) replacement were main plots and the biochar treatments, hardwood, softwood, and control (no biochar) were randomized as subplots. Each treatment combination was replicated four times accounting for 36 experimental units. The field is comprised of three irrigation zones corresponding to each irrigation treatment with independent irrigation control. Each irrigation zone consisted of 12 plots of 7.6 m length and 8 m width. The plots within the irrigation zone were separated by 0.9 m wide alleys.

The irrigation application was based on the ET_*c*_ requirement calculated as a product of reference evapotranspiration (ET_*o*_) and stage-specific crop coefficients (*K*_*c*_). The ET_*o*_ was computed from the weather data using the Penman–Monteith method (Zotarelli et al., 2010). The weather data were recorded by a weather station (Davis instruments 6152, Wireless Vantage Pro2, Davis Instruments Corporation, Hayward, California) installed near the experimental site. The *K*_*c*_ values for the sweet corn were used as *K*_*c*_ initial = 0.40 [0–20 days after planting (DAP)], *K*_*c*_ crop development = 0.80 (20–45 DAP), *K*_*c*_ mid = 1.15 (45–70 DAP), *K*_*c*_ late = 1.00 (70–80 DAP) (Brouwer and Heibloem, 1986). The irrigation water needs were calculated as a difference of ET_*c*_ and precipitation. Irrigation was applied once a week to restore the ET_*c*_ for the previous week. A water meter was installed for each zone to measure the volume of applied water. The electrical conductivity (EC) and pH of irrigation water were 2.2 mmhos/cm and 7.65, respectively.

### Soil Sampling and Analyses

At the beginning of the experiment in 2019, the core and bulk soil samples were collected from the experimental field within the 0–30 cm depth before the biochar application and planting. The samples were analyzed to determine the physical properties at 0–30 cm depth (10 cm increment) while the chemical properties were determined for 0–10 cm depth (Table 2). The bulk soil samples were air-dried at room temperature and crushed to pass through a 2 mm sieve. The particle size analysis was conducted using the hydrometer method (Gee and Bauder, 1986) and USDA textural classification (Soil Science Division Staff, 2017).

**TABLE 2:** Soil’s physical and chemical properties in 2019, Lubbock, TX.

Property	Unit	Soil depth		
		0–10 cm	10–20 cm	20–30 cm
% Sand	%	61.8	60.8	59.3
% Silt	%	27.1	29.1	30.9
% Clay	%	11.1	10.1	9.8
Bulk density	g cm^–3^	1.461	1.749	1.728
Saturated water content (0 kPa)	% v/v	0.499	0.392	0.394
Field capacity (-33 kPa)	% v/v	0.223	0.225	0.226
Permanent wilting point (-1,500 kPa)	% v/v	0.157	0.191	0.192
Organic matter %	%	0.8	-	-
pH		7.5	-	-
NO3 N	ppm	3	-	-
Phosphorus	ppm	38	-	-
Potassium	ppm	488	-	-

To assess the effect of biochar treatments on soil properties, soil core and bulk samples were collected from each experimental plot at the end of the growing season in 2020. The bulk soil samples were collected with an auger at 0–10 cm depth from three sample points in each plot. For determining the soil pH and EC, bulk samples were air-dried and ground to pass through a 2 mm sieve. Soil (15 g) was suspended in the deionized water in a 1:1 (w/w; soil/deionized water) ratio using 50 ml conical tubes. The samples in the conical tubes were mixed thoroughly using the Eberbach’s benchtop fixed-speed reciprocal shaker for 15 min and left overnight at room temperature. The samples were centrifuged at 10,000 rpm for 5 min to collect the supernatant aqueous solution in another set of fresh conical tubes. After calibration with multiple standard solutions for the instrument, the soil EC and pH were measured using the Orion Star pH/Conductivity Portable Meter in the aqueous solution.

The undisturbed core soil samples were collected in 5 cm × 5 cm stainless steel cores at 0–5 and 5–10 cm soil depths. The cores were pushed into the soil using a core sampler with a slide hammer (AMS, Inc.). The measurements of the soil water retention curves, that is the functional relationship between the soil matric potential and soil water content, were made on these core samples under 7 different pressures (0 (saturation), -10, -33, -250, -500, -1,000, and -1,500 kPa) following the pressure plate apparatus procedure described by Saini et al. (2020). The soil bulk density was determined using the core method (Blake and Hartge, 1986) and calculated as the ratio of the dry soil mass (oven-dried at 105°C for 24 h) to the bulk soil volume (volume of the core). The soil porosity was calculated by dividing the volume of water held at saturation by the bulk soil volume (volume of the core). The plant available water (PAW) was calculated as the difference between the soil water content at field capacity (at -33 kPa) and the permanent wilting point (at -1,500 kPa), which were obtained from the soil water retention curve.

### Gas Exchange and Chlorophyll

The physiological responses of sweet corn to irrigation and biochar treatments were assessed by measuring the *g*_*s*_, E, and *P*_*n*_ using a portable photosynthesis system (Model LI-6800, LI-COR Biosciences, Lincoln, NE, United States). All physiological measurements were recorded using two young fully expanded leaves from two randomly chosen plants within each experimental plot. The portable photosynthesis system was used at a steady state by keeping 1,500 μmol m^–2^ s^–1^ photosynthetic active radiation (PAR), 400 μmol mol^–1^ reference CO_2_ concentration, 700 μmol^–1^ air flow rate, 65% of relative humidity, and switching off the temperature control. These gas exchange measurements were done at 37, 60, and 80 DAP in 2019 and 38, 56, and 81 DAP in 2020. The iWUE was calculated as the ratio of *P*_*n*_ and *g*_*s*_. The chlorophyll was determined using the SPAD 502 Plus chlorophyll meter (Spectrum Technologies, Inc.). The chlorophyll measurements were recorded at 30, 52, and 80 DAP in 2019 and 24, 55, and 81 DAP in 2020. The physiological observations were recorded between 10:00 and 14:00 h because the plants were well-lighted and fully active during this time.

### Growth and Yield

The leaf area index (LAI) was measured using a ceptometer (Model: AccuPAR LP-80, Decagon Devices Inc.) from two sites per plot during solar noon. The ceptometer was placed close to the plant base parallel to the rows. The instrument was calibrated based on the coordinates of the experimental location and date. The device measured the PAR and intercepted the PAR non-destructively and used an in-built equation to calculate the LAI.

A total of five plants were selected randomly in each plot to measure the plant height from the soil surface to the tip of the tassels at 80 DAP in 2019 and 69 DAP in 2020. An area of 7.6 m^2^ was hand-harvested from each plot on 31 July (87 DAP) in 2019 and 10 August (87 DAP) in 2020 to determine the ear yield and total fresh plant biomass. The representative ear and plant samples from each plot were weighed and oven-dried at 70°C to a constant weight to determine the moisture content. The resulting moisture content was used to calculate the ear and total plant dry weights.

The harvest index (HI) was calculated using equation (1).


(1) $$\mathrm{HI}=\frac{\mathrm{Fresh}\,\mathrm{ear}\,\mathrm{yield}}{\mathrm{Total}\,\mathrm{aboveground}\,\mathrm{fresh}\,\mathrm{weight}}$$

The WP was calculated using equation (2).


(2) $$\mathrm{WP}=\frac{\mathrm{Fresh}\,\mathrm{ear}\,\mathrm{yield}}{\mathrm{Irrigation}+\mathrm{Rainfall}}$$

### Statistical Analysis

We conducted the analysis of variance (ANOVA) with a split-plot design in the R version 3.5.2 using the Agricolae package version 1.2-8 to analyze the collected data. Data were analyzed separately for each year. The least significant difference (LSD) test at a 5% significance level was used to compare the treatment means. The SigmaPlot software version 14 (Systat Software, San Jose, CA) was used to make figures.

## Results

### Atmospheric Conditions and Irrigation

**FIGURE 1:**
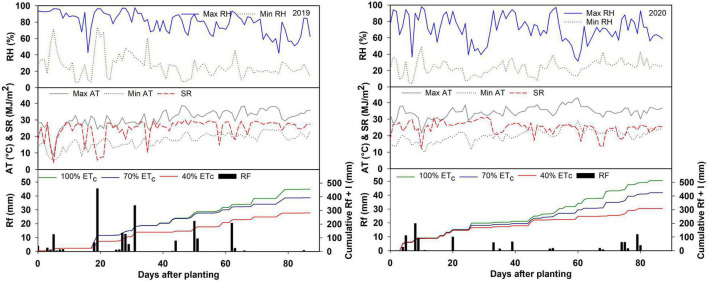
Daily maximum (max) and minimum (min) relative humidity (RH), daily maximum and minimum air temperature (AT), daily average solar radiation (SR), rainfall (Rf), and cumulative rainfall plus irrigation (RF + I) during the 2019 and 2020 growing seasons.

The prevailing weather conditions during the sweet corn growing season in 2019 and 2020 are described in Figure 1. The average relative humidity was 53.2 % in 2019 and 47.3% in 2020. The average temperature during the growing season was 24.3 and 27.3°C in 2019 and 2020, respectively. Overall, the 2020 growing season was drier and hotter compared to 2019. In 2020, trial plants were exposed to hot and dry spells at 50–65 DAP (Figure 1). The daily average solar radiation was recorded as 24.7 MJm^–2^ in 2019 and 24.9 MJ/m^–2^ in 2020. As an output of the above-described weather conditions, the reference evapotranspiration (ET_*o*_) in 2020 was higher compared to 2019 (Table 3). The total rainfall during the growing season was 203 mm in 2019 and 101 mm in 2020. Therefore, the irrigation requirements in 2020 were substantially higher than in 2019 (Table 3). All the plots received an equal amount of initial irrigation to ensure good crop establishment. In 2019, the irrigation treatments began 43 days after planting due to continuous rainfall in the early season (Figure 1). In 2020, the irrigation treatments were started at 26 DAP.

**TABLE 3:** Irrigation amount, rainfall, reference evapotranspiration (ET_*o*_), and water-saving for irrigation treatments in the 2019 and 2020 growing season.

Irrigation treatments	Irrigation amount (mm)		Rainfall (mm)		ET_*o*_		% water-saving	
	2019	2020	2019	2020	2019	2020	2019	2020
100% ET_*c*_	252	412	203	101	572	614	–	–
70% ET_*c*_	189	323	203	101	572	614	24	22
40% ET_*c*_	78	208	203	101	572	614	69	50

### Biochar Effects on the Soil Properties

**FIGURE 2:**
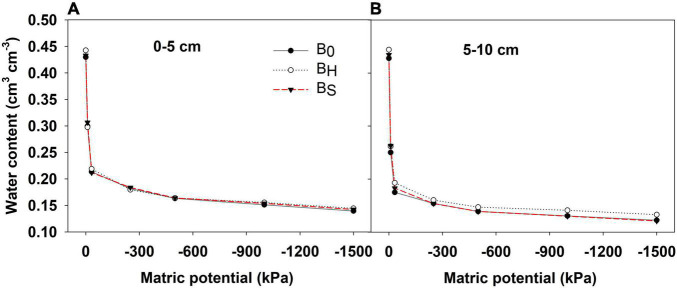
Measured soil water retention curves at **(A)** 0–5 cm and **(B)** 5–10 cm soil depths for biochar treatments after harvest in 2020.

**FIGURE 3:**
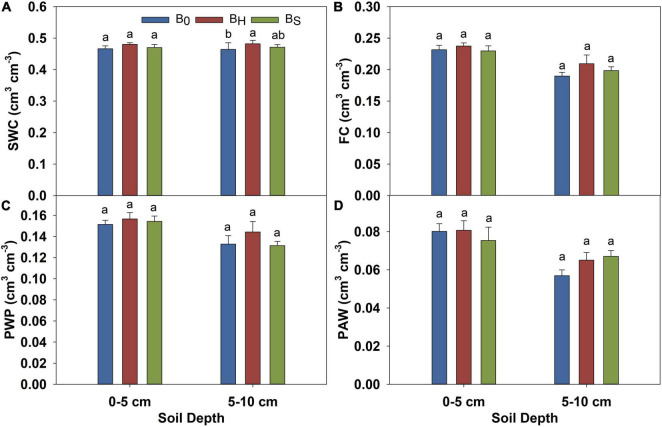
Effect of biochar on the volumetric water content of soil at **(A)** saturated water content (0 kPa), **(B)** field capacity (-33 kPa), **(C)** the permanent wilting point (-1,500 kPa), and **(D)** plant available water after harvest in 2020. The error bars represent ± standard error. Different letters indicate significant differences (*p* ≤ 0.05) among treatments.

The biochar application did not affect the soil pH and EC (Table 4). Hardwood biochar application reduced the soil bulk density by 4% at 0–5 cm depth and improved soil porosity over non-amended plots by 3.9% at 5–10 cm soil depth (Table 4). However, softwood biochar did not affect the bulk density and porosity. The measured volumetric water content was higher in the hardwood biochar treatment than softwood and non-amended plot at all pressures. The magnitude of the differences was greater at 5–10 cm soil depth (Figure 2). As shown in Figure 3, the hardwood biochar soil samples held more water at saturation, field capacity, and permanent wilting point, but the differences were significant only for the saturated water content. The plant available water was higher in the biochar treatments compared to the non-amended plots but the differences were non-significant.

**TABLE 4:** Effect of biochar treatments on soil pH, electrical conductivity (EC), bulk density, and porosity.

	EC (μS cm^–1^)	pH	Bulk density (g cm^–3^)		Porosity	
Soil depth →	0–10 cm	0–10 cm	0–5 cm	5–10 cm	0–5 cm	5–10 cm
Control (B_0_)	1,139 a	8.14 a	1.51 a	1.52 a	0.466 a	0.464 b
Hardwood (B_*H*_)	1,147 a	8.16 a	1.45 b	1.49 a	0.480 a	0.482 a
Softwood (B_*S*_)	1,018 a	8.11 a	1.51 a	1.51 a	0.470 a	0.471 ab

*Different letters within a column indicate significant differences (P ≤ 0.05) among treatments.*

### Deficit Irrigation and Biochar Effects on the Physiological Parameters

**FIGURE 4:**
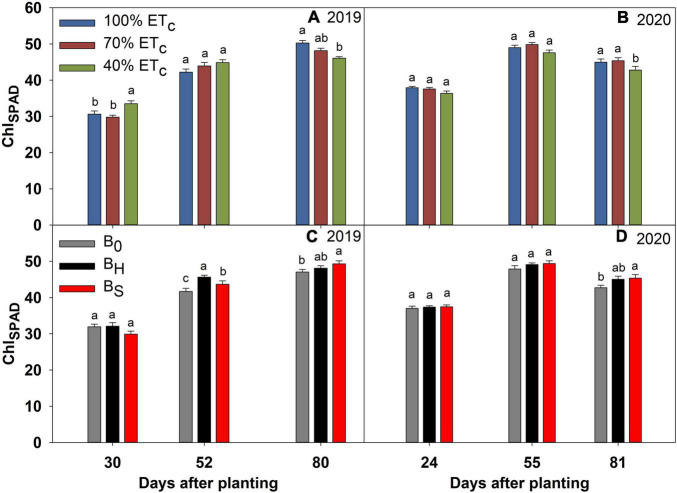
Chlorophyll (Chl_*SPAD*_) of sweet corn under deficit irrigation **(A,B)** and biochar application **(C,D)** during the 2019 and 2020 growing seasons. The error bars represent ± standard error. Different letters indicate significant differences (*p* ≤ 0.05) among treatments on a measurement day in Figures 4–8.

**FIGURE 5:**
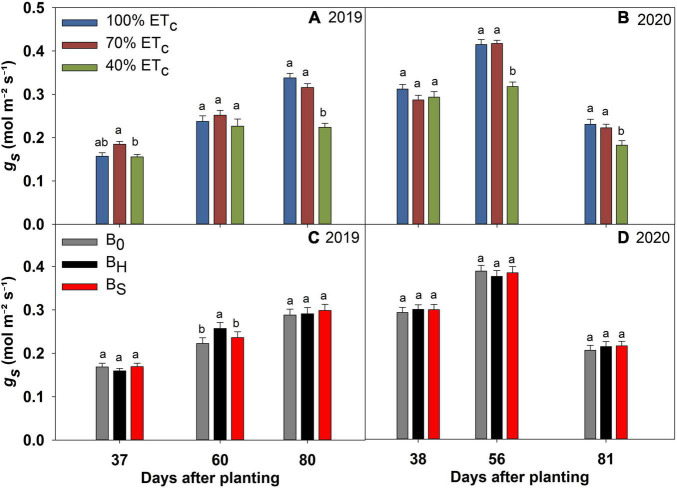
Stomatal conductance (*g*_*s*_) of sweet corn under deficit irrigation **(A,B)** and biochar application **(C,D)** during the 2019 and 2020 growing seasons.

**FIGURE 6:**
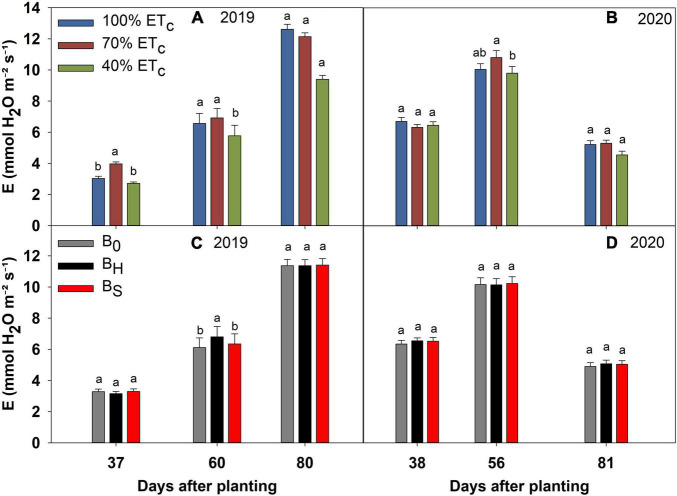
Transpiration (E) of sweet corn under deficit irrigation **(A,B)** and biochar application **(C,D)** during the 2019 and 2020 growing seasons.

**FIGURE 7:**
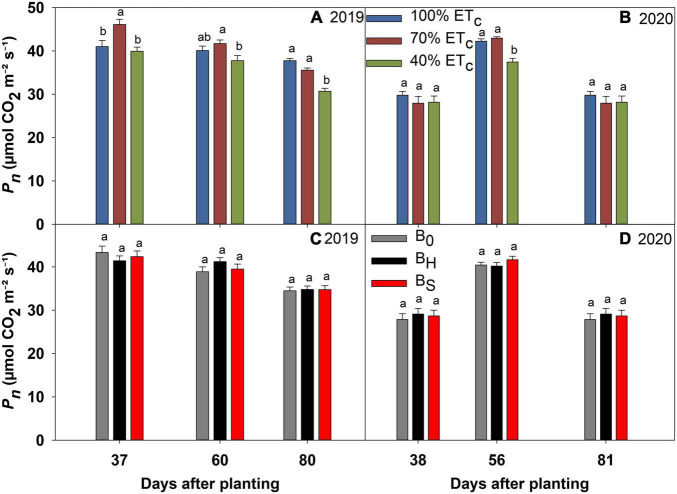
Photosynthesis (*P*_*n*_) of sweet corn under deficit irrigation **(A,B)** and biochar application **(C,D)** during the 2019 and 2020 growing seasons.

**FIGURE 8:**
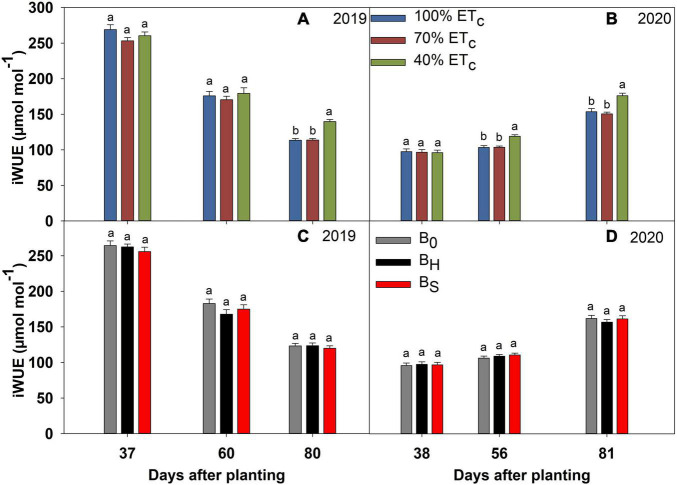
Intrinsic water use efficiency (iWUE) of sweet corn under deficit irrigation **(A,B)** and biochar application **(C,D)** during the 2019 and 2020 growing seasons.

The effect of DI and biochar on Chl_*SPAD*_, *g*_*s*_, E, *P_*n*_*, and iWUE of sweet corn is presented in Figures 4–8. The interactions among irrigation and biochar treatments were non-significant for all the measured physiological parameters. The differences in Chl_*SPAD*_ among the irrigation treatments became significant toward the end of the growing season in both years (Figure 4). In both years, the Chl_*SPAD*_ recorded under 100% ET_*c*_ at 80 DAP was at par with 70% ET_*c*_ but significantly greater than 40% ET_*c*_. Both biochar treatments increased the Chl_*SPAD*_ over non-amended during both years (Figure 4). The differences in Chl_*SPAD*_ due to the biochar treatments were significant at 52 and 80 DAP in 2019 and 81 DAP in 2020.

The 40% ET_*c*_ treatment reduced the *g*_*s*_ significantly at 80 DAP in 2019, and 56 and 81 DAP in 2020 compared to other irrigation treatments (Figure 5). Consequently, the plants in the 40% ET_*c*_ also recorded a significant reduction in E compared to 100% ET_*c*_ during both years (Figure 6). The *P*_*n*_ responses to the irrigation treatments followed a similar pattern as E during both years but the magnitude of the decline in *P*_*n*_ due to DI was much lower compared to E (Figure 7). For instance, compared to 100% ET_*c*_, the E under 40% ET_*c*_ was reduced by 14 and 36% at 60 and 80 DAP in 2019, respectively. However, the decrease of 9 and 14% in *P*_*n*_ under the 40% ET_*c*_ on the same days was much lower than the decrease in E. Consequently, the iWUE under 40% ET_*c*_ increased by 23 and 15% compared to 100% ET_*c*_ in 2019 and 2020, respectively, toward the end of the growing season (Figure 8). The 70% ET_*c*_ maintained a statistically similar *g*_*s*_, E, *P*_*n*_, and iWUE as 100% ET_*c*_ during both years. The biochar treatments did not have any significant effect on the gas exchange parameters during both years except at 60 DAP in 2019 when the plants in the hardwood biochar treatment recorded significantly higher *g*_*s*_ (Figure 5) and E compared to the control plots (Figure 6).

### Deficit Irrigation and Biochar Effects on Plant Growth and Yield

The interactions among the irrigation and biochar treatments for the measured plant growth and yield parameters were non-significant during both years. In 2019, 40% ET_*c*_ decreased the plant height significantly whereas the height of 70% ET_*c*_ plants was comparable to 100% ET_*c*_ (Table 5). In 2020, both DI treatments (70 and 40% ET_*c*_) decreased the plant height significantly by 5 and 16 cm compared to full irrigation (100% ET_*c*_). The biochar treatments did not have a significant effect on the plant height in 2019 but the hardwood biochar increased the plant height significantly over the non-amended plots in 2020. The irrigation and biochar treatment had a non-significant effect on LAI in both years.

**TABLE 5:** Effect of deficit irrigation and biochar application on growth and yield parameters, and water productivity (WP) of sweet corn.

Treatment	Plant height	Leaf area index	Aboveground vegetative dry biomass	Total aboveground dry biomass	Yield	Harvest index	WP
							
	cm		kg ha^−1^	kg ha^−1^	kg ha^−1^	(%)	kg ha^–1^mm^–1^
**2019**							
**Irrigation (I)**							
100% ET_*c*_	138.0 a	4.61 a	1,165 ab	3,450 b	12,648 a	65.1 a	27.8 b
70% ET_*c*_	139.0 a	4.61 a	1,350 a	4,239 a	13,726 a	64.1 a	35.0 a
40% ET_*c*_	130.8 b	4.29 a	1,027 b	3,122 b	10,527 b	66.5 a	37.6 a
**Biochar (B)**							
B_0_	134.3 a	4.42 a	1,114 a	3,409 a	12,075 a	65.5 a	33.0 a
B_*H*_	136.6 a	4.63 a	1,234 a	3,700 a	12,427 a	65.3 a	33.8 a
B_*S*_	136.8 a	4.45 a	1,194 a	3,702 a	12,398 a	64.8 a	33.7 a
Interaction (I × B)	ns	ns	ns	ns	ns	ns	ns
**2020**							
**Irrigation (I)**							
100% ET_*c*_	143.4 a	4.62 a	2,804 a	5,230 a	7,707 a	39.7 a	15.0 b
70% ET_*c*_	138.3 b	4.79 a	2,460 b	4,830 a	7,495 a	40.7 a	17.7 a
40% ET_*c*_	127.1 c	4.30 a	2,371 b	3,663 b	4,320 b	31.4 b	14.0 b
**Biochar (B)**							
B_0_	134.3 b	4.49 a	2,428 b	4,434 a	6,461 a	37.9 a	15.4 a
B_*H*_	137.8 a	4.78 a	2,645 a	4,716 a	6,626 a	37.0 a	15.9 a
B_*S*_	136.8 ab	4.44 a	2,561 ab	4,573 a	6,435 a	37.0 a	15.4 a
Interaction (I × B)	ns	ns	ns	ns	ns	ns	ns

*Different letters in a column within a factor indicate significant differences (P ≤ 0.05) among treatments. ‘ns’ represents non-significant difference/interaction.*

The 70% ET_*c*_ produced the highest aboveground vegetative dry biomass and total aboveground dry biomass followed by 100 and 40% ET_*c*_ in 2019 (Table 5). However, in 2020, these values were in the order of 100, 70, and 40% ET_*c*_. In 2020, both DI treatments reduced the aboveground vegetative dry biomass significantly but the 70% ET_*c*_ produced comparable total aboveground dry biomass as 100% ET_*c*_. The biochar treatments had a non-significant effect on these parameters in 2019, but the hardwood biochar application significantly increased the aboveground vegetative dry biomass compared to the control in 2020. The 40% ET_*c*_ decreased the ear yield by 17%, whereas 70% ET_*c*_ increased the yield by 9% compared to 100% ET_*c*_ in 2019 (Table 5). In 2020, the ear yield values in 70 and 40% ET_*c*_ were 3 and 44% lower than 100% ET_*c*_, respectively. The biochar treatments did not affect the ear yield during both years. On average, the ear yield in 2020 dropped by 47% compared to 2019. However, the total aboveground dry biomass was higher, and the aboveground vegetative dry biomass was more than double in 2020 compared to 2019. The HI differences due to the irrigation treatments were non-significant in 2019. However, 100 and 70% ET_*c*_ treatments recorded significantly higher HI compared to 40% ET_*c*_ in 2020 (Table 5). The biochar treatments did not affect HI during both years.

In 2019, 70 and 40% ET_*c*_ treatments improved the WP significantly over 100% ET_*c*_ by 26 and 35%, respectively. However, in 2020, the 70% ET_*c*_ treatment improved the WP by 18% whereas 40% ET_*c*_ reduced the WP by 7% compared to 100% ET_*c*_. The biochar treatments did not affect the WP during both years. Overall, the WP was approximately double in 2019 than in 2020.

## Discussion

### Effect of Biochar on Soil pH and Water Retention Characteristics

Biochar acts as a liming agent and often increases the soil pH due to its alkaline nature. However, we did not observe the effect of biochar on soil pH, which was expected because the pH of both biochars used in this study was comparable to the soil pH. The biochar application generally decreases the bulk density of soils and increases the porosity with a greater effect in coarse-textured soils (Blanco-Canqui, 2017). Our results indicate a 4% decrease in the bulk density at 0–5 cm soil depth with 13 Mg ha^–1^ hardwood biochar application, but no biochar effect at 5–10 cm depth in the sandy clay loam soil profile. Zheng et al. (2016) reported a 4 and 7 % decrease in the bulk density of sandy loam soil with 20 and 40 Mg ha^–1^ application of wheat straw biochar, respectively. However, Rogovska et al. (2016) observed no change in the bulk density after the hardwood biochar application at 9.9 and 18.4 Mg ha^−1^ in loam, clay loam, and silty clay loam soil. An increase in the soil porosity at 5–10 cm soil depth with hardwood biochar application resulted in enhanced soil water retention characteristics in the hardwood plots compared to control (Figure 2). However, the statistical analyses indicated that the hardwood biochar effect was significant only for saturated water content. Notably, saturated water content provided a measure of the total porosity of the soil. The differences in the soil water content at field capacity, permanent wilting point, and plant available water among the biochar treatments were non-significant. The effect of the biochar application at different rates on soil water retention has yielded contrasting results in different studies. For instance, similar to our results, Moragues-Saitua et al. (2017) also observed no increase in water retention with *Miscanthus* sp. biochar application (10 and 20 Mg ha^–1^) on sandy loam soil. Contrarily, Ma et al. (2016) observed a significant increase in the field capacity and plant available water with 7.8 Mg ha^–1^ application of maize straw and peanut hull biochar.

The investigation of the irrigation levels and application of two types of biochar in sweet corn revealed that the interactive effects were non-significant for any of the examined parameters. The irrigation levels affected all the parameters significantly whereas the biochar effects were significant only on Chl_*SPAD*_, plant height, and vegetative dry biomass. The results of this study and their implications for sweet corn production in semi-arid climate are discussed with a focus on the main effects.

### Sweet Corn Physiology, Biomass, and Yield as Affected by Deficit Irrigation

Water stress generally has an adverse effect on the gas exchange, plant growth, and biomass but its effect on chlorophyll content is conflicting. Some researchers report an increase (Ramírez et al., 2014; Gavili et al., 2019) while others report a decrease (Mansouri-Far et al., 2010; Langeroodi et al., 2019), and still, others observe no change (Pandey et al., 2000) in the chlorophyll levels under water stress. The increased chlorophyll concentration due to moisture stress is mainly attributed to the increased concentration of nitrogen (N) and magnesium (Mg) with a corresponding reduction in the plant biomass due to the essential roles these elements play in chlorophyll synthesis (Gavili et al., 2019). However, our results indicate a significant drop in Chl_*SPAD*_ at higher water stress (40% ET_*c*_) only toward the end of the growing season. This agrees with the results of Mansouri-Far et al. (2010) who observed a significant decline in the leaf chlorophyll content by withholding irrigation at the reproductive stage (R3) of maize. Brevedan and Egli (2003) also observed a decline in the chlorophyll levels in soybean exposed to water stress at the seed filling stage. The imposition of water deficit causes a reduction in the uptake of N and Mg leading to a reduction in chlorophyll synthesis and its concentration in the leaves (Peuke and Rennenberg, 2011; Bista et al., 2018). Nevertheless, Chl_*SPAD*_ under mild water stress of 70% ET_*c*_ remained comparable to full irrigation (100% ET_*c*_). Langeroodi et al. (2019) also observed a minimal decline in the chlorophyll content index at mild water stress of 60% maximum allowable depletion (MAD) of available water compared to 45% MAD but reported a significant drop with further increase in water stress.

In response to water stress, plants close stomata to reduce transpiration losses. The reduction in *g*_*s*_ means reduced gas exchange and a consequent decrease in CO_2_ assimilation (Chaves et al., 2002). In this study, a significant decline in *P*_*n*_ under 40% ET_*c*_ indicates that E losses were prevented by stomatal closure at the expense of CO_2_ intake. The reduction in *P*_*n*_ decreased the biomass accumulation and yield in 40% ET_*c*_ compared to 100% ET_*c*_. Higher *g*_*s*_ values do not necessarily mean greater *P*_*n*_ and can be less water-efficient (Álvarez and Sánchez-Blanco, 2013). The decreased *g*_*s*_ in response to water stress limiting E losses may still be high enough to maintain satisfactory internal CO_2_ levels (Langeroodi et al., 2019), and thus more water-efficient. Our results indicate that the magnitude of *P*_*n*_ reduction under 40% ET_*c*_ was lower than the reduction in *g*_*s*_ and E, thus achieving higher iWUE with 40% ET_*c*_ compared to 100% ET_*c*_. The 70% ET_*c*_ maintained similar *g_*s*_, P_*n*_*, and E as 100% ET_*c*_ during the whole growing seasons of both years indicating that sweet corn plants can adapt to mild water stress without adverse effects on physiology. As a result, the total aboveground dry biomass and ear yield were not significantly different between 100 and 70% ET_*c*_. Furthermore, both DIs (40 and 70% ET_*c*_) maintained comparable LAI as 100% ET_*c*_ but the reduction in the plant height was significant for 70% ET_*c*_ in 2020 and 40% ET_*c*_ in both years. It may be attributed to a reduction in the internodal length or increased biomass allocation toward the leaves under water deficit to maintain high-net photosynthesis. Kirda et al. (2005) reported that the effect of 50% DI on the maize plant height and LAI was non-significant. Ertek and Kara (2013) reported a 3.8, 14, and 23% decline in fresh ear yield of sweet corn reducing the water use by 30, 45, and 60%, respectively, based on ET_*c*_. Hirich et al. (2012) suggested that a 25% water deficit during the vegetative stage can maintain the sweet corn productivity whereas Oktem (2008) recorded a significant reduction in the yield at a 20% water deficit during the growing season. Our results suggest that 70% ET_*c*_ can be used for sweet corn production in West Texas with minimal yield reduction whereas 40% ET_*c*_ reduces the yield significantly. The WP improved significantly under 70% ET_*c*_ due to the 62 and 89 mm irrigation water-saving without any significant yield reductions over 100% in 2019 and 2020, respectively. The WP for 40% ET_*c*_ improved over full irrigation in 2019 due to a 175-mm reduction in water use and improvement in iWUE. Although the iWUE for 40% ET_*c*_ was significantly higher than the 100% ET_*c*_ in 2020, it did not result in a high WP due to the following reasons.

The weather conditions during the growing season in 2020 characterized by 3°C higher average air temperature, 3% lower average RH, and 103 mm lower rainfall were more stressful compared to 2019. In 2020, the crop experienced a hot and dry spell at 50–65 DAP, which coincided with the anthesis and pollination events. This had an adverse effect on the kernel formation confirmed by the presence of numerous empty kernels in many ears. The high temperatures during flowering inhibit pollen viability and germination, which may cause kernel abortion (Bakhtavar et al., 2015; Li and Howell, 2021). Previous studies have reported heat stress sensitivity of kernel formation in maize and a reduction in the kernel number due to high temperatures around silking ultimately reducing the yield (Niu et al., 2021; Wang et al., 2021). Consequently, a major reduction in yield occurred in 2020 compared to 2019. The adverse effect on yield was more pronounced in 40% ET_*c*_, wherein heat stress was accompanied by higher water stress. The reduction in yield caused higher biomass partitioning toward the vegetative parts resulting in higher vegetative dry biomass and lower HI in 2020 than 2019. Farré and Faci (2009) observed a reduction in HI of maize only when the flowering stage was exposed to water stress. Compared to the other irrigation treatments, the HI dropped significantly under more stressed 40% ET_*c*_ in 2020 due to a greater reduction in the yield caused by poor kernel formation. This resulted in a significant decline in WP of 40% ET_*c*_ in 2020. Overall, sweet corn accumulated 1,260 heat units in 2019 and 1,524 in 2020. The higher heat units resulted in higher total aboveground dry biomass accumulation in 2020.

### Sweet Corn Physiology, Biomass, and Yield as Affected by Biochar Application

The biochar application was expected to alleviate the adverse effect of water stress on the sweet corn physiology, growth, and yield, and improve WP by improving the soil properties. However, our hypothesis was only partially true. The hardwood biochar application decreased soil bulk density and increased porosity, but it did not increase PAW. The hardwood biochar increased the Chl_*SPAD*_, improved *g*_*s*_ and E at mid-stage in 2019, but did not affect the *P*_*n*_ significantly. The plant height and vegetative biomass were improved by the biochar application but it did not have much impact on the yield and WP. These results are in consensus with Vaccari et al. (2015) who reported an improvement in the tomato plant growth with biochar application without any fruit yield gains. Ramlow et al. (2019) reported an increase in soil moisture with woody biochar without alleviating the effect of water stress. Nevertheless, several reports illustrating the potential of biochar to mitigate water stress in crops are available in the literature (Faloye et al., 2019; Singh et al., 2019; Alfadil et al., 2021).

The increased Chl_*SPAD*_ due to biochar application may be attributed to the increased N and Mg concentrations in the leaves. The higher concentrations of N and Mg in hardwood biochar compared to softwood biochar may have resulted in greater Chl_*SPAD*_ values for hardwood biochar at most of the measurement days. Although we did not measure the nutrient concentration in the leaves, chlorophyll concentration is closely correlated with the N concentration and used as a tool to determine the N status of the plants (Oppong Danso et al., 2020).

The increase in the vegetative dry biomass may correspond to the enhanced nutrient uptake by plants due to biochar application, though the differences were significant only in 2020, wherein hardwood biochar plots produced significantly higher vegetative dry biomass compared to control. Langeroodi et al. (2021) reported an increased concentration of N, P, K, and Mg in the sunflower (*Helianthus annuus* L.) shoots by paper sludge biochar application. The concentrations of most macro- and micronutrients were higher in hardwood biochar compared to softwood (Table 1). Based on the 2-year data, the hypothesis that biochar may improve the water status of the sweet corn plants under water stress was hardly true as it did not alter the gas exchange parameters significantly. Although hardwood biochar application increased the *g*_*s*_ and E at mid-stage during 2019, it did not cause a significant improvement in the *P*_*n*_ and ear yield.

## Conclusion

This research investigated the implications of using biochar under water-limited conditions in a semi-arid environment for enhancing water and food sustainability. Soil amendment with hardwood biochar marginally affected the physical properties of soil by decreasing bulk density and improving saturated water content, but it did not improve plant available water significantly. The moderate DI (70% ET_*c*_) was found to be the most water-efficient among irrigation treatments. This moderate reduction in water use maintained the plant physiology, growth, and yield similar to 100% ET_*c*_ for 2 consecutive years. The hardwood biochar application at the rate of 13 Mg ha^–1^ increased the chlorophyll content, plant height, and vegetative dry biomass, marginally affecting the gas exchange but did not alter the yield and WP. The results of this 2-year study suggest that biochar application provided only limited benefits for sweet corn production under DI. However, for better understanding, its potential benefits under limited irrigation in the long run, its effect on chemical and biological properties along with crop production need to be investigated in long-term studies. The 70% ET_*c*_ can be recommended as an alternative to 100% ET_*c*_ for water-efficient sweet corn production in West Texas.

## Data Availability Statement

The original contributions presented in the study are included in the article/supplementary material, further inquiries can be directed to the corresponding author/s.

## Author Contributions

SS and MS contributed to the conception and design of the study. MS performed the experiment, analyzed the data, prepared figures, and wrote the manuscript. VP helped in data collection. SS supervised the study. SS, GR, SKD, and RWW provided guidance on data collection and revised the manuscript. All authors approved the submitted manuscript.

## Conflict of Interest

The authors declare that the research was conducted in the absence of any commercial or financial relationships that could be construed as a potential conflict of interest.

## Publisher’s Note

All claims expressed in this article are solely those of the authors and do not necessarily represent those of their affiliated organizations, or those of the publisher, the editors and the reviewers. Any product that may be evaluated in this article, or claim that may be made by its manufacturer, is not guaranteed or endorsed by the publisher.
